# Oral Administration of Ginseng Ameliorates Cyclosporine-Induced Pancreatic Injury in an Experimental Mouse Model

**DOI:** 10.1371/journal.pone.0072685

**Published:** 2013-08-29

**Authors:** Sun Woo Lim, Kyoung Chan Doh, Long Jin, Shang Guo Piao, Seong Beom Heo, Yu Fen Zheng, Soo Kyung Bae, Byung Ha Chung, Chul Woo Yang

**Affiliations:** 1 Convergent Research Consortium for Immunologic Disease, The Catholic University of Korea, Seoul, Korea; 2 Transplant Research Center, The Catholic University of Korea, Seoul, Korea; 3 Division of Nephrology, Department of Internal Medicine, Seoul St. Mary’s Hospital, The Catholic University of Korea, Seoul, Korea; 4 College of Pharmacy, The Catholic University of Korea, Seoul, Korea; 5 College of Pharmacy, Seoul National University. Seoul, Korea; 6 Nephrology and Dialysis Unit, Department of Internal Medicine, YanBian University Hospital, Jilin, China; Omaha Veterans Affairs Medical Center, United States of America

## Abstract

**Background:**

This study was performed to investigate whether ginseng has a protective effect in an experimental mouse model of cyclosporine-induced pancreatic injury.

**Methods:**

Mice were treated with cyclosporine (30 mg/kg/day, subcutaneously) and Korean red ginseng extract (0.2 or 0.4 g/kg/day, oral gavage) for 4 weeks while on a 0.01% salt diet. The effect of ginseng on cyclosporine-induced pancreatic islet dysfunction was investigated by an intraperitoneal glucose tolerance test and measurements of serum insulin level, β cell area, macrophage infiltration, and apoptosis. Using an in vitro model, we further examined the effect of ginseng on a cyclosporine-treated insulin-secreting cell line. Oxidative stress was measured by the concentration of 8-hydroxy-2′-deoxyguanosine in serum, tissue sections, and culture media.

**Results:**

Four weeks of cyclosporine treatment increased blood glucose levels and decreased insulin levels, but cotreatment with ginseng ameliorated the cyclosporine-induced glucose intolerance and hyperglycemia. Pancreatic β cell area was also greater with ginseng cotreatment compared with cyclosporine monotherapy. The production of proinflammatory molecules, such as induced nitric oxide synthase and cytokines, and the level of apoptotic cell death also decreased in pancreatic β cell with ginseng treatment. Consistent with the in vivo results, the in vitro study showed that the addition of ginseng protected against cyclosporine-induced cytotoxicity, inflammation, and apoptotic cell death. These in vivo and in vitro changes were accompanied by decreases in the levels of 8-hydroxy-2′-deoxyguanosine in pancreatic β cell in tissue section, serum, and culture media during cotreatment of ginseng with cyclosporine.

**Conclusions:**

The results of our in vivo and in vitro studies demonstrate that ginseng has a protective effect against cyclosporine-induced pancreatic β cell injury via reducing oxidative stress.

## Introduction

The development of cyclosporine (CsA) opened a new era in transplantation medicine [1]. However, its adverse effects, such as nephrotoxicity, hypertension, dyslipidemia, and glucose intolerance, often give rise to considerable metabolic derangement. In particular, posttransplant diabetes mellitus (PTDM) has emerged as a major adverse event, occurring in 10–25% of the patients receiving immunosuppressive therapy [2], [3]. This condition often leads to serious consequences, including reduced graft survival and increased risk of infectious and cardiovascular diseases, which confer significant morbidity and mortality. The pathogenesis of PTDM is thought to be partly related to the direct toxic effect of CsA on pancreatic β-cells and the consequent reduction in insulin synthesis and secretion [4], [5]. Recent studies demonstrated that CsA-induced oxidative stress can play a pivotal role in pancreatic islet dysfunction, including hyperglycemia, reduced insulin level and pancreatic islet mass, and increased apoptosis and macrophage infiltration, because CsA produces free radical species in the pancreas [6]–[11].

Panax ginseng Carl Anton Von Meyer (C.A. Meyer) has been used widely used as a traditional remedy in oriental medicine. Red ginseng is Panax ginseng that has been heated, steamed and dried. As a consequence of this process, Panax ginseng undergoes certain biochemical changes and acquires particular physiological activities, including free radical-scavenging, vasodilating, and antidiabetic properties [12]–[14]. A recent study found that ginsenoside Re could be used as an effective antidiabetic agent because it restored antioxidant activity in streptozotocin-induced diabetic rats [15].

However, the antioxidative effect of ginseng in CsA-induced pancreatic islet β cell injury has not been studied. Therefore, we investigated the effects of ginseng on CsA-induced oxidative stress in chronic CsA toxicity. To define this, we evaluated pancreatic β cell function, islet size, macrophage infiltration, and apoptosis in a well-known experimental mouse model of chronic CsA toxicity. The antioxidant effect of ginseng was examined using the expression of 8-hydroxy-2′-deoxyguanosine (8-OHdG), which is a marker of oxidative damage to DNA. Our data clearly demonstrate that administration of ginseng has a protective effect against CsA-induced pancreatic islet β cell injury via reducing oxidative stress.

## Materials and Methods

### Animals and Drugs

The Animal Care and Use Committee of the Catholic University of Korea approved the experimental protocol (permit CUMC-2012-0069-01), and all procedures performed in this study followed ethical guidelines for animal studies. All surgery was performed with the animals anesthetized with Zoletil 50 (10 mg/kg, intraperitoneally; Virbac Laboratories, Carros, France) and Rompun (15 mg/kg, intraperitoneally, Bayer, Leuverkusen, Germany), and all efforts were made to minimize suffering. Eight-week-male ICR (Institute for Cancer Research) mice weighing 25–30 g (Taconic Anmed, Rockville, MD) were housed in individual cages in a temperature- and light-controlled environment. They were fed a low-salt diet (0.01% sodium; Teklad Premier Laboratory Diets, Madison, WI) with tap water ad libitum. CsA (100 mg/mL) provided by Novartis Pharma (Basel, Switzerland) was diluted in olive oil (Sigma-Aldrich, St. Louis, MO) to a final concentration of 30 mg/kg. Korean red ginseng extract (KRG) obtained from Korea Ginseng Corporation (Seoul, Korea) was diluted in sterile water. According to the manufacturer’s data, the main components of the Korean red ginseng extract are Rg1 (2.01%), Rb1 (8.27%), Rg3 (s) (1.04%), Re (2.58%), Rc (3.90%), Rb2 (3.22%), Rd (1.09%), Rf (1.61%), Rh1 (0.95%), and Rg2 (s) (1.35%).

### Experimental Design

Mice were randomized into eight groups of 10 animals per group, as follows.

1. Vehicle (VH) group: mice received a daily subcutaneous injection of olive oil (5 mL/kg) and oral administration of sterile water for 4 weeks.
2. VH+K0.2 group: mice received a daily subcutaneous injection of olive oil (5 mL/kg) and oral administration of KRG (0.2 mg/kg) for 4 weeks.
3. VH+K0.4 group: mice received a daily subcutaneous injection of olive oil (5 mL/kg) and oral administration of KRG (0.4 mg/kg) for 4 weeks.
4. CsA group: mice received a daily subcutaneous injection of CsA (30 mg/kg) and oral administration of sterile water for 4 weeks.
5. CsA+K0.2 group: mice received a daily subcutaneous injection of CsA (30 mg/kg) and oral administration of KRG (0.2 mg/kg) for 4 weeks.
6. CsA+K0.4 group: mice received a daily subcutaneous injection of CsA (30 mg/kg) and oral administration of KRG (0.4 mg/kg) for 4 weeks.

The dosage and route of administration for CsA in mice were chosen based on a previous study [16], [17].

### Basic Protocol

Mice were randomly assigned to different treatment groups. Body weight was monitored daily. For 24 hr before euthanasia, animals were individually housed in metabolic cages (Tecniplast, Gazzada, Italy) for 24 h urine collections. On the following day, animals were anesthetized with Zoletil 50 (10 mg/kg, intraperitoneally; Virbac Laboratories) and Rompun (15 mg/kg, intraperitoneally; Bayer) to minimize suffering. Blood samples were obtained by orbital bleeding. After blood collection, tissues were harvested for further analysis.

### Measurement of Whole Blood or Tissue Concentrations of CsA

Pancreatic tissues from 3–4 mice were pooled to reach approximately 1 g, rinsed with cold saline to eliminate blood remaining in the tissue, and homogenized with 4 volumes of saline using a Polytron homogenizer (Kinematica AG, Lucerne, Switzerland). After centrifugation, the supernatant fraction was quantified using liquid chromatography-tandem mass spectrometry (LC-MS/MS) using an API 3000 LC-MS/MS machine (Applied Biosystems, Foster City, CA) equipped with an electrospray ionization interface to generate negative ions. The basal level of CsA in whole blood was also measured using LC-MS/MS.

### Intraperitoneal Glucose Tolerance Test

The intraperitoneal glucose tolerance test (IPGTT) was performed on day 28. Briefly, after 1 day of fasting, 25% dextrose (2 g/kg) was injected, and the blood glucose concentration was measured just before and at 30, 60, 90, and 120 min after the injection using a glucose analyzer (Accu-Check, Roche Diagnostics, Basel, Switzerland). The area under the curve of glucose (AUCg) was calculated by trapezoidal estimation from the values obtained in the IPGTT.

### Measurement of Serum Insulin

To measure fasting serum insulin concentration, blood samples were obtained after overnight fasting at the same time as the first IPGTT sample. The serum insulin concentration was measured using a competitive enzyme-linked immunosorbent assay (Shibayagi Co., Gunma, Japan).

### Measurement of 8-hydroxy-2′-deoxyguanosine

Oxidative DNA damage was evaluated by the level of DNA adduct 8-hydroxy-2′-deoxyguanosine (8-OHdG) in serum and conditioned culture media with CsA (25 µM) or CsA plus KRG-treated INS-1 cells using competitive enzyme-linked immunosorbent assay (Cell Biolabs, San Diego, CA).

### Preservation of Pancreas Tissue

Pancreas was preserved by in vivo perfusion through the left ventricle of the heart. Animals were perfused with phosphate-buffered saline (PBS) to flush blood from the tissues, then perfused with periodate–lysine–paraformaldehyde (PLP) solution, and postfixed overnight in PLP at 4°C. After dehydration in a graded series of ethanol, the tissues were embedded in paraffin for immunohistochemistry.

### Measurement of Pancreatic β Cell Area

A minimum of 20 fields per section were assessed using a color image analyzer (TDI Scope Eye™ Version 3.0 for Windows; Olympus, Tokyo, Japan). Briefly, captured images from double immunofluorescence with insulin and glucagon were quantified using the Polygon program by measuring the pancreas area seen to contain insulin-positive area except vacuoles when viewed under×200 magnifications. Histopathologic analysis was performed on randomly selected fields of pancreas section by a pathologist blinded to the identity of the treatment groups.

### Immunohistochemistry for Insulin, Glucagon, F4/80, 8-OHdG, Inducible Nitric Oxide Synthesase, Interleukin-6, Interleukin-17, Bcl-2, Bax and Active Caspase-3

For double immunofluorescence labeling for insulin and glucagon, tissue sections were incubated with a mix of antibodies against insulin (1∶500; Zymed, San Francisco, CA) and glucagon (1∶200; Cell Signaling Technology, Inc. Danvers, MA), washed with PBS and incubated with a mix of Cy^3^-conjugated antibody (1∶1000; Jackson ImmunoResearch Laboratories, West Grove, PA) and fluorescein isothiocyanate (FITC)-conjugated antibody (1∶50; Jackson ImmunoResearch Laboratories). For these studies, specific antibodies against F4/80 (1∶100; Serotec, Oxford, UK), 8-OHdG (1∶100; JaICA, Shizuoka, Japan), iNOS (1∶200; Santa Cruz Biotechnology, Santa Cruz, CA), IL-6 (1∶50; R&D Systems, Minneapolis, MN), IL-17 (1∶50; R&D Systems), Bcl-2 (1∶50; Santa Cruz Biotechnology), Bax (1∶50; Delta Biolabs, Gilroy, CA), active caspase-3 (1∶50; Millipore, Billerica, MA) were incubated on tissue sections or in rat insulin-secreting cell (INS-1). For coloration, sections were incubated with a mixture of 0.05% 3, 3′-diaminobenzidine (DAB) containing 0.01% H_2_O_2_ until a brown color was visible. For double labeling with insulin, tissue sections were washed with PBS and then incubated with an anti-insulin antibody (1∶500; Zymed) and then with a Cy^3^-conjugated antibody (Jackson ImmunoResearch Laboratories). F4/80-positive cells or insulin, Bcl-2, Bax, active caspase-3 and 8-OHdG-positive areas were estimated semiquantitatively using a color image analyzer (TDI Scope Eye™). The sections were examined and photographed using a fluorescence microscope (Axio Imager. M2, Carl Zeiss, Jena, Germany).

### Immunoblot Analysis for iNOS, IL-6, IL-17, Bcl-2, Bax, Caspase-3, and β-actin

Immunoblotting analysis of the whole pancreas was performed as described [16]. INS-1 cells were lysed in RIPA buffer (150 mM NaCl, 1% NP-40, 0.5% sodium deoxycholate, 0.1% SDS, 50 mM Tris, pH 8.0). Immunoblot analysis was processed as described previously [16]. iNOS (1∶200; Santa Cruz Biotechnology), IL-6 (1∶200; R&D Systems), IL-17 (1∶200; R&D Systems), Bcl-2 (1∶200; Santa Cruz Biotechnology), Bax (1∶200; Delta Biolabs), caspase-3 (1∶100; Millipore), and β-actin (1∶20,000; Sigma-Aldrich) were detected by incubating for 12 h with specific antibodies at 4°C. β-actin blots as loading controls are from the same gel as the immunoblot they are paired with. Proteins were visualized with SuperSignal Chemiluminescence Substrate (GE Healthcare Life Science, Buckinghamshire, UK) following the manufacturer’s recommendations. Relative optical densities of bands in each lane were normalized with each β-actin band from the same gel.

### In situ TdT-mediated dUTP-biotin Nick End-labeling Assay

Apoptosis in tissue sections and INS-1 cells was identified using the ApopTag *in situ* apoptosis detection kit (Millipore). For double immunofluorescence labeling for insulin, tissue sections were treated as described above. The numbers of TUNEL-positive cells (stained with DAB) were counted in 20 different fields in each section at 200×magnification.

### In vitro Treatment of INS-1 Cells with CsA and KRG

To investigate the direct effect of KRG on CsA-induced pancreatic β cell injury, we examined apoptosis, oxidative stress and inflammation in INS-1 cell line, which was a gift from Dr. Yoon (Catholic University of Korea, Seoul, Korea). This cell line is the most commonly used clonal cell model in pancreatic β cell research [18]. INS-1 cells were cultured in Roswell Park Memorial Institute (RPMI) 1640 medium (Wisent, Saint-Bruno, QC, Canada) supplemented with 11.1 mM sodium pyruvate, 10 mM Hepes, 10% fetal bovine serum (FBS; Wisent), 2 mM L-glutamine, 50 µM β-mecaptoethanol, 100 U/mL penicillin, and 100 mg/mL streptomycin (all from Sigma-Aldrich). The cells were incubated in a humidified atmosphere of 5% CO_2_, 95% air at 37°C for 24 h and subcultured at 70–80% confluence. For the experiments, we plated INS-1 cells onto dishes in RPMI 1640 medium containing 10% FBS for 24 h and then switched cells to serum-free media containing CsA (25 µM) in the presence or absence of KRG (1 or 10 µg/mL). After 24 h, the cells and culture supernatant were harvested for further analysis. These experiments were conducted at least three times. The experiments were performed with individual samples from separate experiments and not using different wells from the same culture plate.

### Measurement of Nitrite Level in the Culture Medium of CsA- and KRG-treated INS-1 Cells

Accumulation of nitrite, an indicator of nitric oxide (NO) synthesis, was measured in the culture medium using the Griess reagent system (Promega, Madison, WI). Briefly, 100 µL of culture medium was mixed with 100 µL of Griess reagent (equal volumes of 1% w/v naphtylethylenediamine and HCl) and incubated at room temperature for 10 min. Light absorbance at 550 nm was then measured using a microplate reader. Fresh culture medium was used as a blank in all experiments. The amount of nitrite in the test samples was calculated from a sodium nitrite standard curve. These experiments were conducted at least three times with different samples.

### Measurement of Apoptosis in CsA- and KRG- Treated INS-1 Cells using Flow Cytometry

INS-1 cells were incubated in 100 µL binding buffer (10 mM HEPES, pH 7.4, 150 mM NaCl, 5 mM KCl, 1.8 mM CaCl_2_, 1 mM MgCl_2_) containing 5 µL Allophycocyanin Conjugate (APC)-Annexin V (BD Pharmingen, San Diego, CA) at 25°C in the dark for 15 min. Binding buffer (400 µL) was added to dilute the samples before analysis on a flow cytometer. The cells positively labeled for Annexin V were considered apoptotic. These experiments were conducted three times at least.

### Statistical Analysis

Data are expressed as mean ± standard error of at least three independent experiments. Groups were compared using a one-way analysis of variance with Bonferroni’s correction (SPSS, Chicago, IL). Statistical significance was assumed at P<0.05.

## Results

### Effect of KRG on Pancreatic Function in CsA-induced Pancreatic Injury

**Figure 1:**
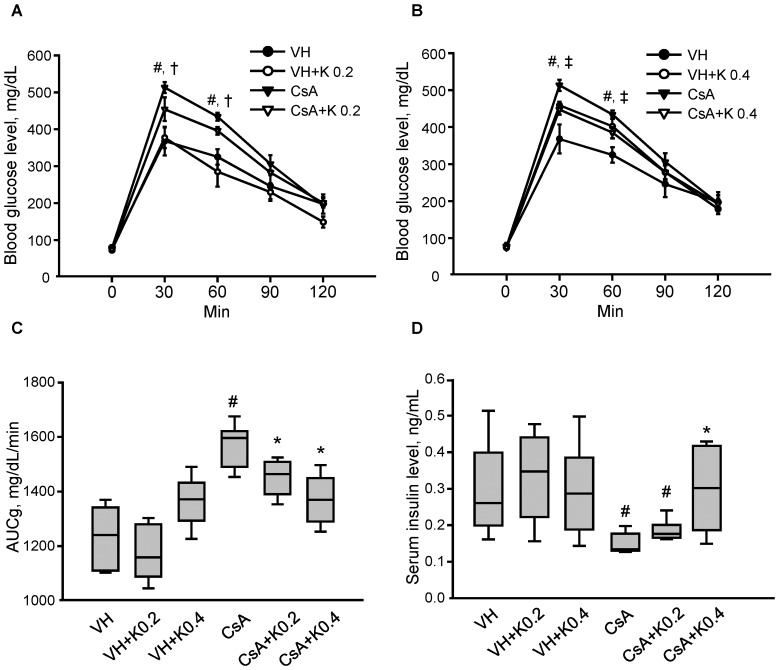
Effect of KRG on pancreatic function in CsA-induced pancreatic injury. (A and B) Results of IPGTT among the experimental groups. CsA-treated mice had significantly elevated blood glucose compared with the VH-only control group at 30 and 60 min. CsA plus daily treatment with 0.2 or 0.4 mg/kg KRG (K0.2 or K0.4, respectively) decreased blood glucose levels significantly at 30 and 60 min compared with the CsA-only group. (C) Calculation of the AUCg for the experimental groups. CsA plus K0.2 or K0.4 treatment resulted in a decreased AUCg compared with the CsA-only group. (D) Serum insulin levels in the experimental groups. The CsA plus K0.2 or K0.4 groups had significantly increased insulin levels compared with the CsA-only group. Values are mean ± standard errors (SE: *n* = 10). ^#^P<0.05 vs. VH; *P<0.05 vs. CsA; ^†^P<0.05 vs. CsA+K0.2; ^‡^P<0.05 vs. CsA+K0.4.

Table 1 shows the effects of 4 weeks of CsA and KRG (K) treatment on the basic parameters of the experimental groups. Both CsA+K groups (one treated daily with 0.2 g/kg and one with 0.4 g/kg of KRG) showed lower serum creatinine (Scr) and blood urea nitrogen (BUN) than the CsA only group. KRG cotreatment did not affect the CsA level in either whole blood or pancreatic tissues, indicating that drug interactions did not occur at these doses. Baseline blood glucose levels did not differ among the four groups (Figure 1A and B). However, the blood glucose level after intraperitoneal glucose loading was significantly higher in the CsA group than in the VH group, but cotreatment with KRG significantly decreased the blood glucose level at 30 and 60 min after glucose loading compared with the CsA group. KRG cotreatment with CsA decreased the calculated area under the curve for glucose (AUCg) compared with the CsA group (Figure 1C: VH, 1229±55 mg/dL/min; VH+K0.2, 1174±49 mg/dL/min; VH+K0.4, 1360±35 mg/dL/min; CsA, 1563±39 mg/dL/min; CsA+K0.2, 1447±32 mg/dL/min; CsA+K0.4, 1368±44 mg/dL/min; VH vs. CsA, CsA vs. CsA+K0.2 or CsA+K0.4, P<0.05). The fasting insulin level was significantly lower in the CsA group than in the VH group. It was significantly increased in both CsA+K groups compared with the CsA group (Figure 1D: VH, 0.30±0.05 ng/mL; VH+K0.2, 0.33±0.05 ng/mL; VH+K0.4, 0.30±0.04 ng/mL; CsA, 0.15±0.01 ng/mL; CsA+K0.2, 0.19±0.01 ng/mL; CsA+K0.4, 0.30±0.06 ng/mL; VH vs. CsA, CsA vs. CsA+K0.2 or CsA+K0.4, P<0.05). Thus, oral KRG administration during CsA-induced pancreatic dysfunction in mice improved glucose tolerance and restored defective insulin secretion, suggesting that KRG has an antihyperglycemic effect.

**Table 1:** Effect of KRG on basic parameters.

	VH	VH+K0.2	VH+K0.4	CsA	CsA+K0.2	CsA+K0.4
Body weight, g	37±1.0	36±1.0	37±0.2	37±0.6	36±0.5	38±0.7
Urine volume, mL	0.7±0.2	0.8±0.2	0.9±0.3	1.9±0.5*	2.0±0.6	1.7±0.3*
Scr, mg/dL	0.23±0.02	0.22±0.03	0.25±0.01	0.38±0.08*	0.20±0.02#	0.21±0.02#
BUN, mg/dL	13.9±0.5	15.0±0.2	14.1±0.8	15.9±0.7*	13.5±0.8#	13.0±0.9#
Blood CsA conc., ng/mL	–	–	–	1470±61	1399±86	1464±163
Pancreas CsA conc., ng/g	–	–	–	1576±148	1455±55	1447±101

VH, vehicle; K and KRG, Korean red ginseng; CsA, cyclosporine A. Scr, serum creatinine; BUN, blood urea nitrogen. Values are SE (*n* = 10).

* P<0.05 versus VH,

# P<0.05 versus CsA.

### Effect of KRG on Pancreatic β Cell Area in CsA-induced Pancreatic Injury

**Figure 2:**
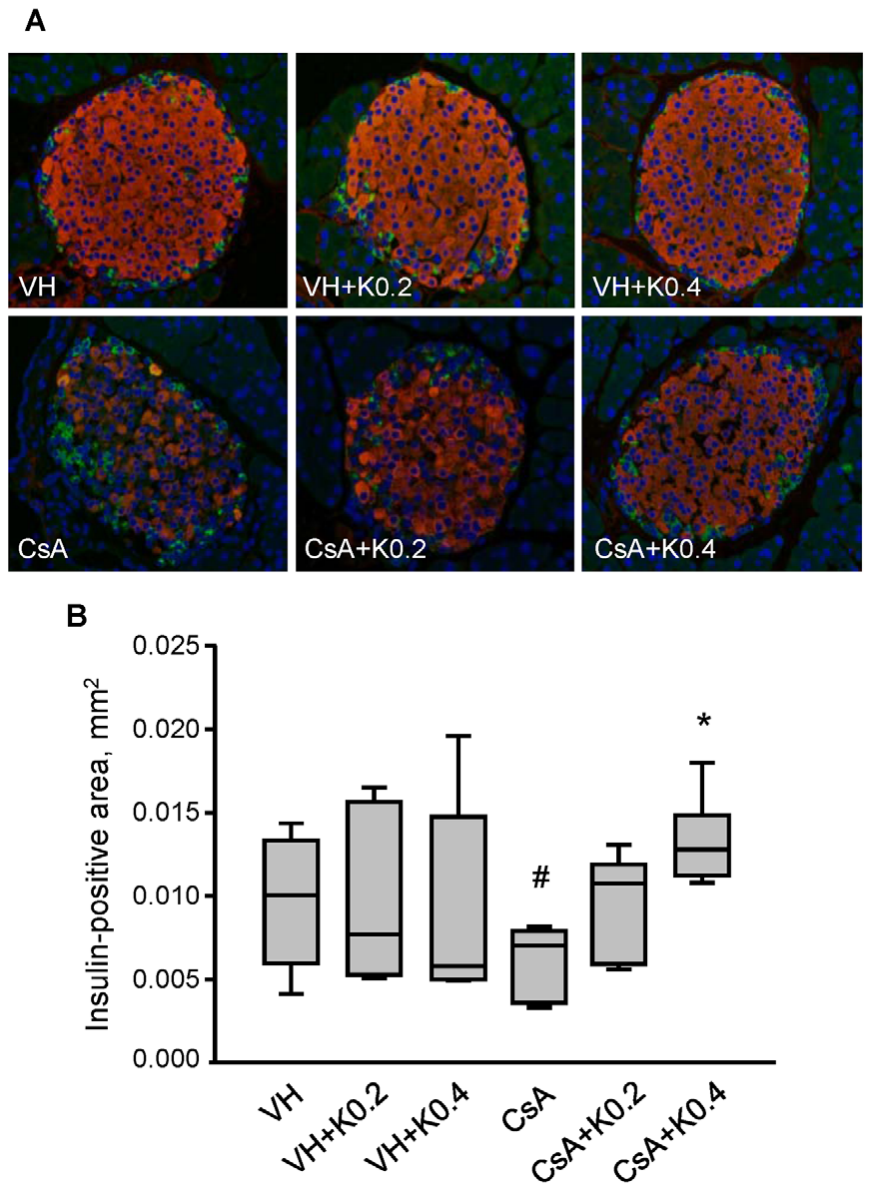
Effects of KRG on pancreatic islet morphology and cell area in CsA-induced pancreatic injury. (A) Double immunostaining for insulin (red) and glucagon (green) showing pancreatic islet morphology and size in the experimental groups. Well-preserved pancreatic islet structure and insulin-immunoreactive areas were seen in the groups treated with VH alone and with VH plus KRG. CsA treatment reduced the insulin-positive area and caused marked cellular vacuolization. However, CsA plus K0.2 or K0.4 treatments resulted in increased insulin immunoreactivity with less pronounced vacuolization, but the latter pathology was still present. (B) Quantitative analysis of total islet area evaluated by counting the insulin-positive areas lacking vacuolization per square millimeter of the pancreatic tissues in the experimental groups. The CsA plus K0.2 or K0.4 groups showed significantly increased insulin-positive areas compared with the CsA-only group. Values are means ± SE (*n* = 10). Magnifications×400. ^#^P<0.05 vs. VH; *P<0.05 vs. CsA.

Double immunofluorescence for insulin (red fluorescence) and glucagon (green fluorescence) in the VH group and VH+K groups showed a strong and uniform pattern of staining in a large proportion of the islet β cells (Figure 2A). By contrast, we observed a lower intensity of insulin staining, irregularity in islet boundaries, and evidence of vacuolization in islet cells in the CsA group. However, both of the CsA+K groups showed increased immunoreactivity for insulin with less pronounced vacuolization than in the CsA group. Compared with the VH group, some cellular vacuoles remained in these groups. Quantification (Figure 2B) showed that the insulin-positive area-except for all vacuoles-in the CsA group was significantly lower than in the VH group (VH, 0.010±0.001/mm^2^; VH+K0.2, 0.010±0.002/mm^2^; VH+K0.4, 0.009±0.002/mm^2^; CsA, 0.006±0.001/mm^2^; VH or VH+K0.2 or VH+K0.4 vs. CsA, P<0.05). Cotreatment with KRG and CsA recovered the insulin-positive area compared with the CsA group (CsA, 0.006±0.001/mm^2^; CsA+K0.2, 0.010±0.001/mm^2^; CsA+K0.4, 0.013±0.001/mm^2^; CsA vs. CsA+K0.4, P<0.05). These results indicate that KRG exerted a significant preservative effect on pancreatic islet β cell in CsA-induced pancreatic injury.

### Effect of KRG on Macrophage Infiltration in CsA-induced Pancreatic Injury

**Figure 3:**
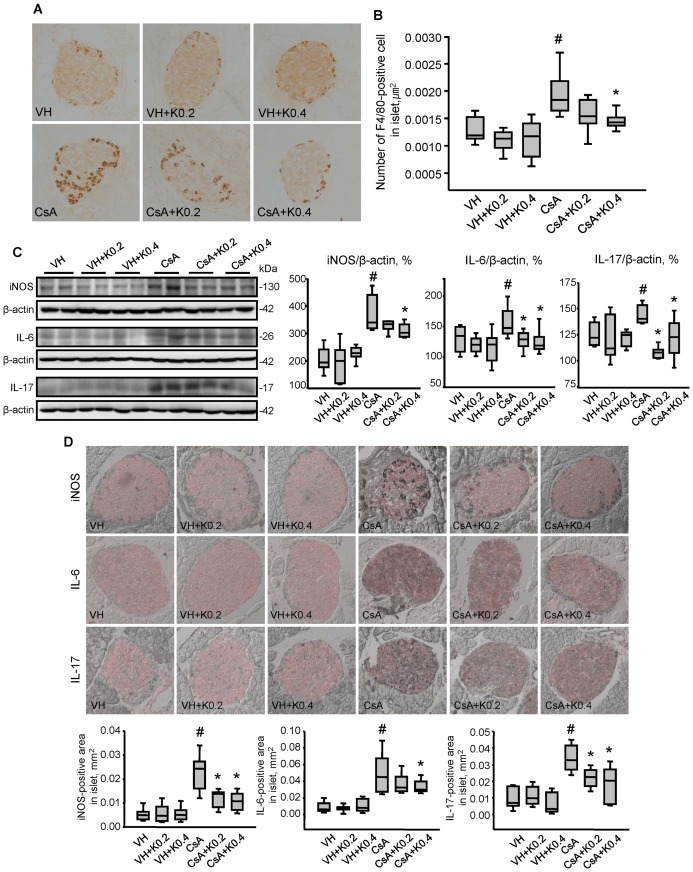
Effect of KRG on pancreatic macrophage infiltration in CsA-induced pancreatic injury. (A) Immunohistochemistry for F4/80, a macrophage marker, on pancreatic islets from the experimental groups. Weak immunoreactivity for F4/80 was found in the groups treated with VH alone or with VH plus KRG. However, the intensity of F4/80 staining increased in the CsA-only group, but this increase was attenuated in the CsA plus K0.2 or K0.4 groups. (B) Quantitative analysis of F4/80-positive islet cells in the experimental groups. The numbers of F4/80-positive islet cells were reduced significantly in the CsA plus K0.2 or K0.4 groups compared with the CsA-only group. (C) Quantitative analysis of iNOS, IL-6 and IL-17 using immunoblotting of whole pancreatic tissues from the experimental groups. The expression levels of these factors were significantly increased in the CsA-only group, but these increases were markedly attenuated by KRG cotreatment. (D) Representative photomicrographs showing double labeling for insulin (red) and iNOS or IL-6 or IL-17 (gray) in pancreatic cells in the same tissue sections from the experimental groups. As in whole pancreatic tissues, quantitative analysis results showed that the immunoreactivities of iNOS, IL-6 and IL-17 were increased in the CsA group, but these increases were decreased markedly by KRG cotreatment. Values are means ± SE (*n* = 10). Relative optical densities of bands in each lane were normalized with each β-actin band from the same gel. Magnifications×400. ^#^P<0.05 vs. VH; *P<0.05 vs. CsA.

To evaluate the effect of KRG on inflammatory cell infiltration of the pancreatic islets, we analyzed the level of infiltration of F4/80-positive cells (mature mouse macrophages). As shown in Figure 3A and 3B, F4/80-positive cells were minimal in the VH, VH+K0.2 and VH+K0.4 groups (VH, 0.0013±0.0001/µm^2^; VH+K0.2, 0.0011±0.0002/µm^2^; VH+K0.4, 0.0011±0.0002/µm^2^). Chronic CsA treatment significantly increased the numbers of F4/80-positive cells, but this increase was markedly attenuated by cotreatment with KRG (CsA, 0.0020±0.0002/µm^ 2^; CsA+K0.2, 0.0016±0.0001/µm^2^; CsA+K0.4, 0.0015±0.0001/µm^2^; CsA vs. CsA+K0.4, P<0.05). Next, we performed immunoblot analysis using pancreatic tissue pieces (Figure 3C). The expression of iNOS was higher in pancreatic tissue from the CsA-treated group than in the VH group, but this increase was attenuated by KRG cotreatment (VH, 207±22%; VH+K0.2, 191±34; VH+K0.4, 228±13; CsA, 370±27%; CsA+K0.2, 326±7%; CsA+K0.4, 310±10%; VH vs. CsA, CsA vs. CsA+K0.4, P<0.05). We also examined the changes in expression of IL-6 and IL-17: important inflammatory cytokines produced by infiltrating cells. Chronic CsA treatment induced higher protein levels of IL-6 and IL-17 than in the VH group, but these increases were attenuated when the mice were cotreated with KRG (IL-6: VH, 131±8%; VH+K0.2, 120±6%; VH+K0.4, 115±13%; CsA, 156±9%; CsA+K0.2, 127±7%; CsA+K0.4, 126±8%; VH vs. CsA, CsA vs. CsA+K0.2 or CsA+K0.4, P<0.05; IL-17: VH, 125±5%; VH+K0.2, 122±10%; VH+K0.4, 121±4%; CsA, 144±4%; CsA+K0.2, 108±3%; CsA+K0.4. 122±7%; VH vs. CsA, CsA vs. CsA+K0.2 or CsA+K0.4, P<0.05). KRG treatment alone did not affect inflammatory cell infiltration or cytokine levels compared with the VH group. Next, we examined the expression of those markers in β cell-specific areas using double immunolabeling for insulin (red fluorescence) and markers stained with DAB in the same section. Each panel in Figure 3D is representative islets and graphs of about 20 islets visualized from 10 animals (iNOS: VH, 0.005±0.001 mm^2^; VH+K0.2, 0.006±0.001 mm^2^; VH+K0.4, 0.006±0.001 mm^2^; CsA, 0.023±0.002 mm^2^; CsA+K0.2, 0.012±0.001 mm^2^; CsA+K0.4, 0.010±0.001 mm^2^; CsA vs. CsA+K0.2 or CsA+K0.4, P<0.05; IL-6: VH, 0.009±0.002 mm^2^; VH+K0.2, 0.007±0.001 mm^2^; VH+K0.4, 0.011±0.003 mm^2^; CsA 0.050±0.008 mm^2^; CsA+K0.2 0.036±0.003 mm^2^; CsA+K0.4, 0.033±0.002 mm^2^; CsA vs. CsA+K0.4, P<0.05; L-17: VH, 0.009±0.002 mm^2^; VH+K0.2, 0.011±0.002 mm^2^; VH+K0.4, 0.007±0.002 mm^2^; CsA, 0.034±0.002 mm^2^; CsA+K0.2, 0.022±0.002 mm^2^; CsA+K0.4, 0.018±0.003mm^2^; CsA vs. CsA+K0.2 or CsA+K0.4, P<0.05). As with the results of immunoblot analysis, the immunoreactivities of iNOS, IL-6, and IL-17 were increased in the CsA group, but were markedly decreased by KRG cotreatment (Figure 3D). These results suggest that KRG had an anti-inflammatory effect on CsA-induced pancreatic β cell injury via the suppression of macrophage infiltration and reduced production of proinflammatory cytokines.

### Effect of KRG on Apoptotic Cell Death in CsA-induced Pancreatic Injury

**Figure 4:**
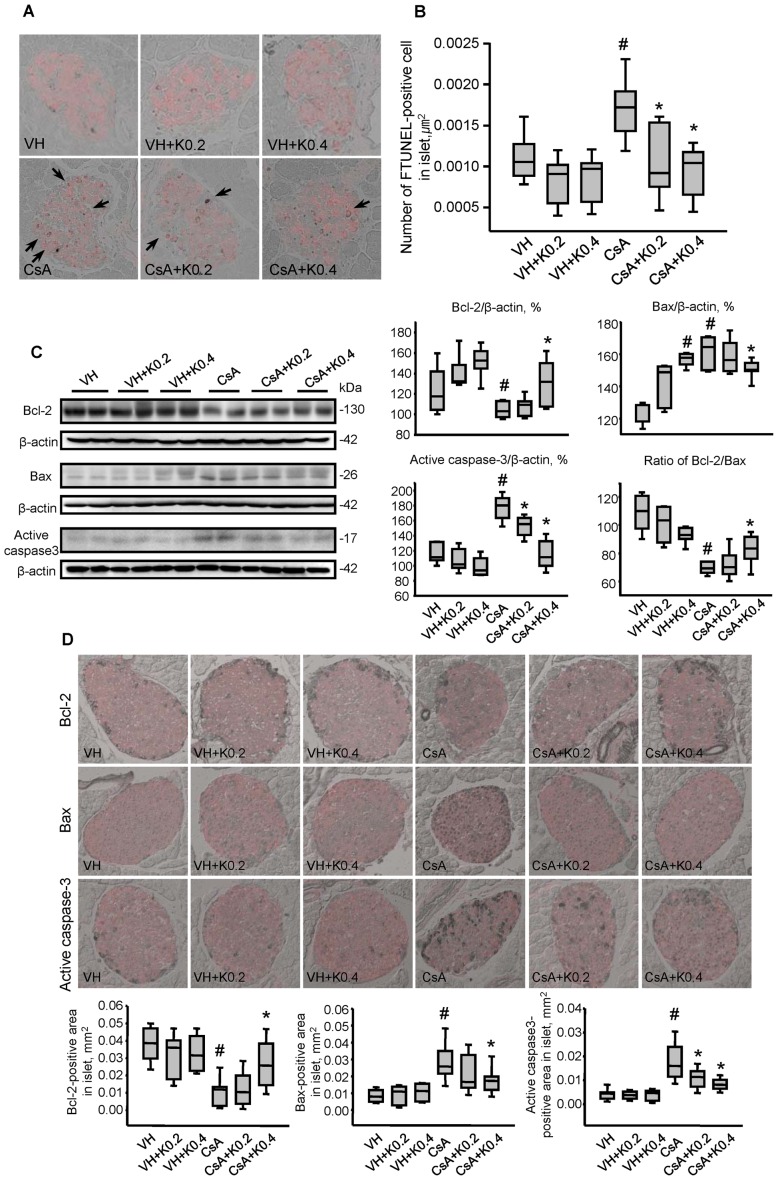
Effect of KRG on apoptotic cell death in CsA-induced pancreatic injury. (A) TUNEL assay for detecting apoptosis in pancreatic cells identified by immunostaining for insulin in the same tissue sections of the experimental groups. Apoptotic cell death featured condensation and fragmentation of the nucleus and shrinkage of the cells with condensation of the cytoplasm and detachment from the surrounding tissues. TUNEL-positive nuclei were rarely observed in the groups treated with VH only or with VH plus K. However, their numbers increased dramatically in the CsA-treated group, but this increase was attenuated in the CsA plus K0.2 or K0.4 groups. (B) Quantitative analysis of TUNEL-positive nuclei in the experimental groups. The numbers were reduced significantly in the CsA plus K0.2 or K0.4 groups compared with the CsA-only group. (C) Quantitative analysis of Bcl-2, Bax and active caspase-3 using immunoblotting of whole pancreatic tissues from the experimental groups. The expression level of Bcl-2, an antiapoptotic marker, was significantly reduced in the CsA plus K0.2 or K0.4 groups compared with the CsA-only group. By contrast, the expression levels of Bax and active caspase-3, proapoptotic markers, were reduced in the CsA plus K0.4 group compared with the CsA-only group. (D) Representative photomicrographs showing double immunolabeling for insulin (red) and Bcl-2 or Bax or active caspase-3 (gray) in pancreatic cells in the same tissue sections from the experimental groups. As in whole pancreatic tissues, quantitative analysis results showed that the immunoreactivity of Bcl-2 was decreased in the CsA group, but this decrease recovered markedly with KRG cotreatment. Bax and active caspase-3 levels were decreased in the CsA plus K-treated groups compared with the CsA group. Values are SE (*n* = 10). Relative optical densities of bands in each lane were normalized with each β-actin band from the same gel. Magnifications×400. ^#^P<0.05 vs. VH; *P<0.05 vs. CsA.

Next, we evaluated whether KRG treatment would modulate apoptotic cell death, an important mechanism of cell death in CsA-induced pancreatic injury [9]. We performed double TUNEL (stained with DAB) and insulin immunostaining (red fluorescence) in the same tissue sections to specify islet β cells, then analyzed the merged images (Figure 4A, B). The numbers of TUNEL-positive cells in islet β cell were significantly higher in the CsA than in the VH groups (VH, 0.0011±0.0001/µm^2^; VH+K0.2, 0.0008±0.0001/µm^2^; VH+K0.4, 0.0008±0.0001/µm^2^; CsA, 0.0017±0.0002/µm^2^; VH vs. CsA, P<0.05). However, coadministration of KRG significantly reduced the number of TUNEL-positive cells in both of the CsA+K groups compared with the CsA group (CsA, 0.0017±0.0002/µm^2^; CsA+K0.2, 0.0011±0.0002/µm^2^; CsA+K0.4, 0.0009±0.0002/µm^2^; CsA vs. CsA+K0.2 or CsA+K0.4, P<0.05). Immunoblot analysis using whole pancreatic tissue pieces also performed to define this. CsA suppressed the expression of the antiapoptotic marker Bcl-2, and this suppression was recovered by cotreatment with KRG. The induction of expression of proapoptotic markers Bax and active caspase-3 by CsA was also attenuated by cotreatment with KRG, as shown in Figure 4C (Bcl-2: VH, 126±9%; VH+K0.2, 141±8%; VH+K0.4, 152±7%; CsA, 105±3%; CsA+K0.2, 107±5%; CsA+K0.4, 132±10; CsA vs. CsA+K0.4, P<0.05; Bax: VH, 124±3%; VH+K0.2, 141±6%; VH+K0.4, 157±2%; CsA, 162±4%; CsA+K0.2, 159±5%; CsA+K0.4, 151±2; CsA vs. CsA+K0.4, P<0.05; active caspase-3: VH, 117±6%; VH+K0.2, 108±7%; VH+K0.4, 99±5%; CsA, 178±7%; CsA+K0.2, 151±5%; CsA+K0.4, 115±9; CsA vs. CsA+K0.2 or CsA+K0.4, P<0.05). There was a significant increase in the Bcl-2/Bax protein ratio in the CsA+K0.4 group compared with the CsA group (1.2-fold higher in the CsA+K0.4 group than the CsA group: VH, 109±6%; VH+K0.2, 101±6%; VH+K0.4, 93±3%; CsA, 70±1%; CsA+K0.2, 73±5%; CsA+K0.4, 83±5; CsA vs. CsA+K0.4, P<0.05). Next, we examined the expression of these markers in islet-specific areas using double immunolabeling for insulin (red fluorescence) and the apoptosis markers (stained with DAB) in the same section (Figure 4D). Each panel in Figure 4D is representative islet and graph of about 20 islets visualized from 10 animals (Bcl-2: VH, 0.038±0.003 mm^2^; VH+K0.2, 0.030±0.004 mm^2^; VH+K0.4, 0.032±0.003 mm^2^; CsA, 0.010±0.002 mm^2^; CsA+K0.2, ±0.003 mm^2^; CsA+K0.4, 0.027±0.005 mm^2^; CsA vs. CsA+K0.4, P<0.05; Bax: VH, 0.008±0.001 mm^2^; VH+K0.2, 0.009±0.002 mm^2^; VH+K0.4, 0.010±0.001 mm^2^; CsA, 0.028±0.003 mm^2^; CsA+K0.2, 0.021±0.003 mm^2^; CsA+K0.4, 0.018±0.002 mm^2^; CsA vs. CsA+K0.4, P<0.05; active caspase-3: VH, 0.004±0.001 mm^2^; VH+K0.2, 0.004±0.001 mm^2^; VH+K0.4, 0.004±0.001 mm^2^; CsA, 0.018±0.002 mm^2^; CsA+K0.2, 0.011±0.001 mm^2^; CsA+K0.4, 0.008±0.001 mm^2^; CsA vs. CsA+K0.2 or CsA+K0.4, P<0.05). As in the whole pancreatic tissues, immunoreactivity of Bcl-2 was decreased in the CsA group, but this decrease was recovered by KRG cotreatment. Bax and active caspase-3 levels decreased in the CsA plus KRG-treated groups compared with the CsA group. This evidence suggests that KRG might protect pancreatic β cells from apoptotic cell death during CsA-induced pancreatic injury.

### Effects of KRG on Oxidative Stress in CsA-induced Pancreatic Injury

**Figure 5:**
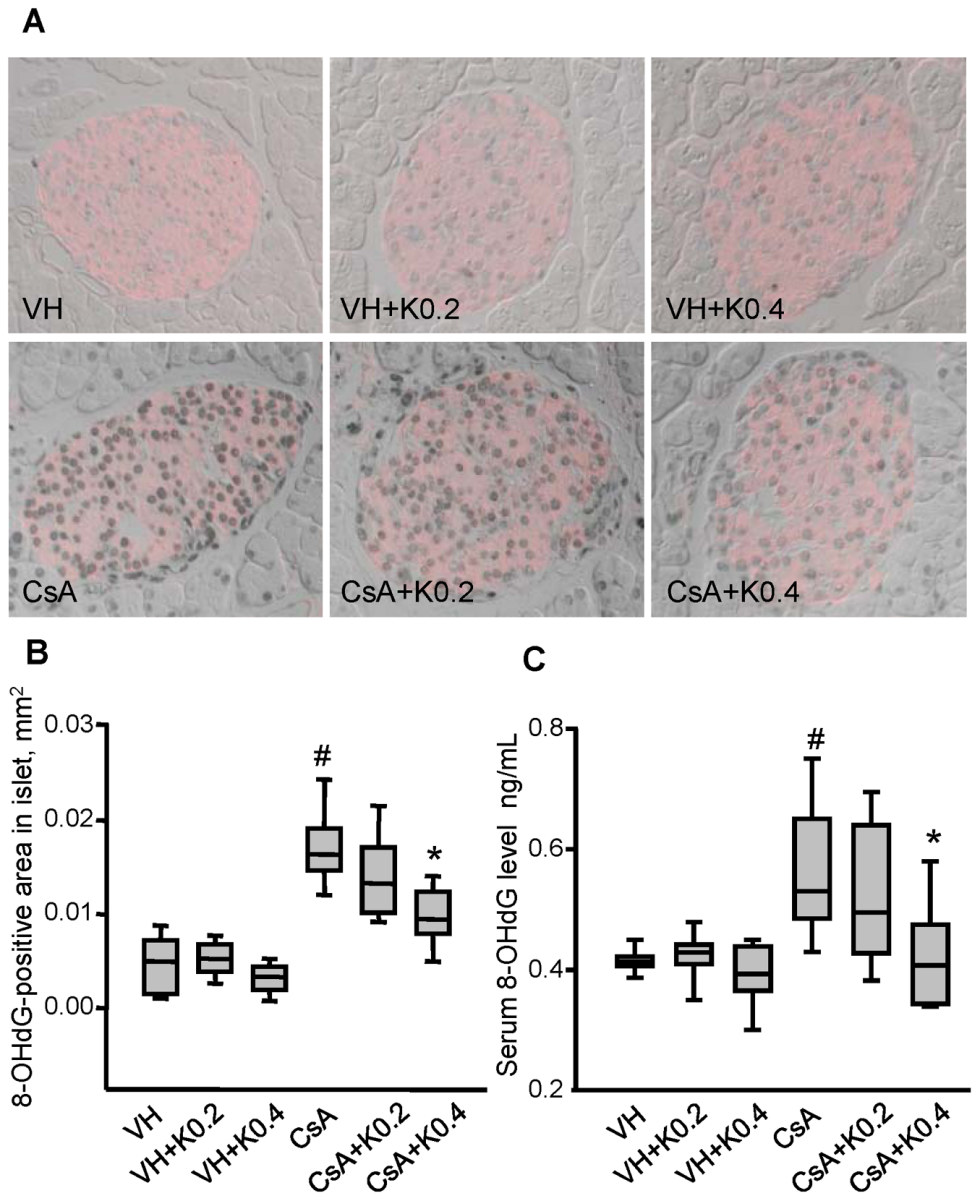
Effect of KRG on oxidative stress in CsA-induced pancreatic injury. (A and B) Double labeling for 8-OHdG (gray) and insulin (red) and quantitative analysis in pancreatic cells in the same tissue sections from the experimental groups. Weak immunoreactivity for 8-OHdG in the nuclei of insulin-positive cells was seen in the VH-alone or VH plus K groups. However, the intensity of 8-OHdG immunostaining increased in the CsA-only group, but this increase was attenuated in the CsA plus K0.2 or K0.4 groups. (C) Changes in the level of serum 8-OHdG in the experimental groups. The CsA plus K0.4 group showed reduced 8-OHdG excretion compared with the CsA-only group. Values are means ± SE (*n* = 10). Magnifications×400. ^#^P<0.05 vs. VH; *P<0.05 vs. CsA.

Oxidative stress is a major cause of chronic CsA toxicity. It results in structural and functional impairment of the kidney and pancreas because CsA produces free radical species in the tissues [8], [16], [19], [20]. To investigate the mechanisms underlying the protective effect of KRG against chronic CsA toxicity, oxidative stress was evaluated by measuring 8-OHdG, which is a reliable marker of cellular oxidative DNA damage. Figure 5A shows that the nuclear 8-OHdG immunoreactivity appeared in the pancreas β cells expressing insulin (red fluorescence). CsA treatment increased the expression of 8-OHdG compared with the VH group, but this increase was attenuated by cotreatment with KRG (Figure 5B: VH, 0.004±0.001 mm^2^; VH+K0.2, 0.005±0.001 mm^2^; VH+K0.4, 0.003±0.001 mm^2^; CsA, 0.017±0.001 mm^2^; CsA+K0.2, 0.014±0.001 mm^2^; CsA+K0.4, 0.010±0.001 mm^2^; CsA vs. CsA+K0.4, P 0.05). CsA treatment also significantly increased the serum level of 8-OHdG compared with the VH group, but cotreatment with KRG attenuated this increase (Figure 5C: VH, 0.42±0.01 ng/mL; VH+K0.2, 0.42±0.02 ng/mL; VH+K0.4, 0.39±0.01 ng/mL; CsA, 0.57±0.04 ng/mL; CsA+K0.2, 0.52±0.03 ng/mL; CsA+K0.4, 0.42±0.03 ng/mL; CsA vs. CsA+K0.4, P<0.05). These findings suggest that oral administration of KRG was associated with improvements in pancreatic β cell function and with reductions in inflammation and apoptotic cell death, by decreasing CsA-induced oxidative stress.

### Effect of KRG on Nitrite Level and Apoptosis in INS-1 Cells during CsA-induced Injury

**Figure 6:**
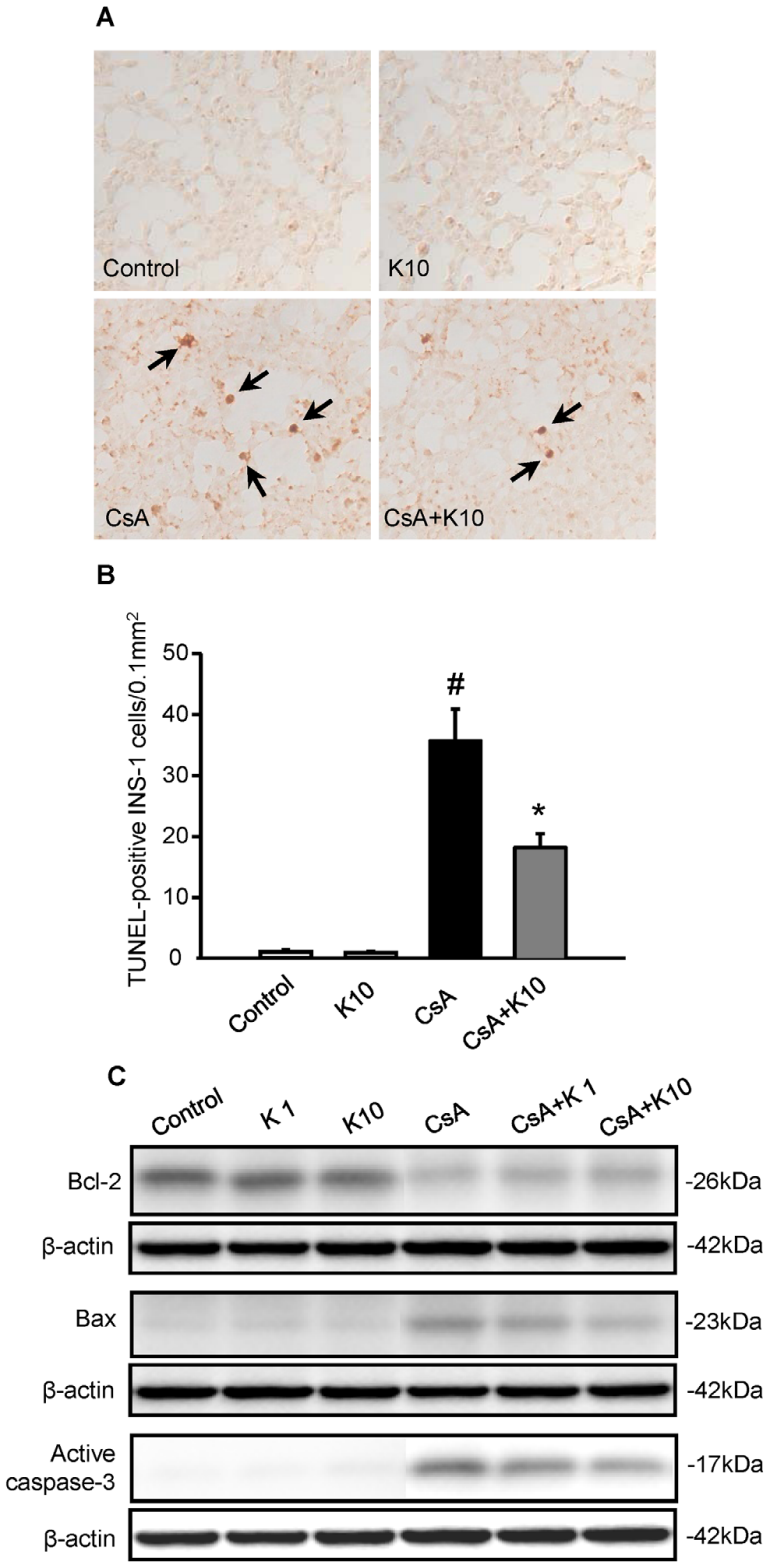
Effect of KRG on nitrite level and apoptosis in INS-1 cells during CsA-induced injury. (A) The level of nitrite, an oxidative product of NO, was markedly lower after cotreatment with CsA plus KRG rather than with CsA alone for 24 h. (B) This graph based on flow cytometric analysis shows annexin V-positive apoptotic cells after cotreatment with CsA plus KRG for 24 h. The percentage of annexin V-positive cells was significantly decreased in cells treated with CsA plus 10 µg/mL of KRG compared with CsA alone. ^#^P<0.05 vs. control; *P<0.05 vs. CsA.

Next, we determined the amount of nitrite, a stable metabolite of NO production in the conditioned media from INS-1 cells treated with CsA alone or with KRG. The nitrite level in media from CsA-treated cells was significantly higher than in the control, and this increase was attenuated by cotreatment with 10 µg/mL of KRG (Figure 6A: 0.34±0.05 µM in the CsA+K10 vs. 0.81±0.14 µM in the CsA, P<0.05). We also evaluated the antiapoptotic effect of KRG during CsA-induced injury in living cells. We performed flow cytometric analysis of APC-annexin V binding, which is a sensitive probe for the identification of cells undergoing apoptosis [21]. Cells were treated with CsA either alone or with KRG (1 or 10 µg/mL). After 24 h of incubation with CsA, 72±3% of CsA-treated cells stained positive for annexin V; however, as shown in Figure 6B, cotreatment with 10 µg/mL of KRG reduced the percentage of Annexin V stained cells to 64±2%. These data further indicate that addition of KRG might protect INS-1 cells from CsA-induced injury by inhibiting NO production and apoptotic cell death.

### Effect of KRG on Apoptotic Cell Death in INS-1 Cells during CsA-induced Injury

**Figure 7:**
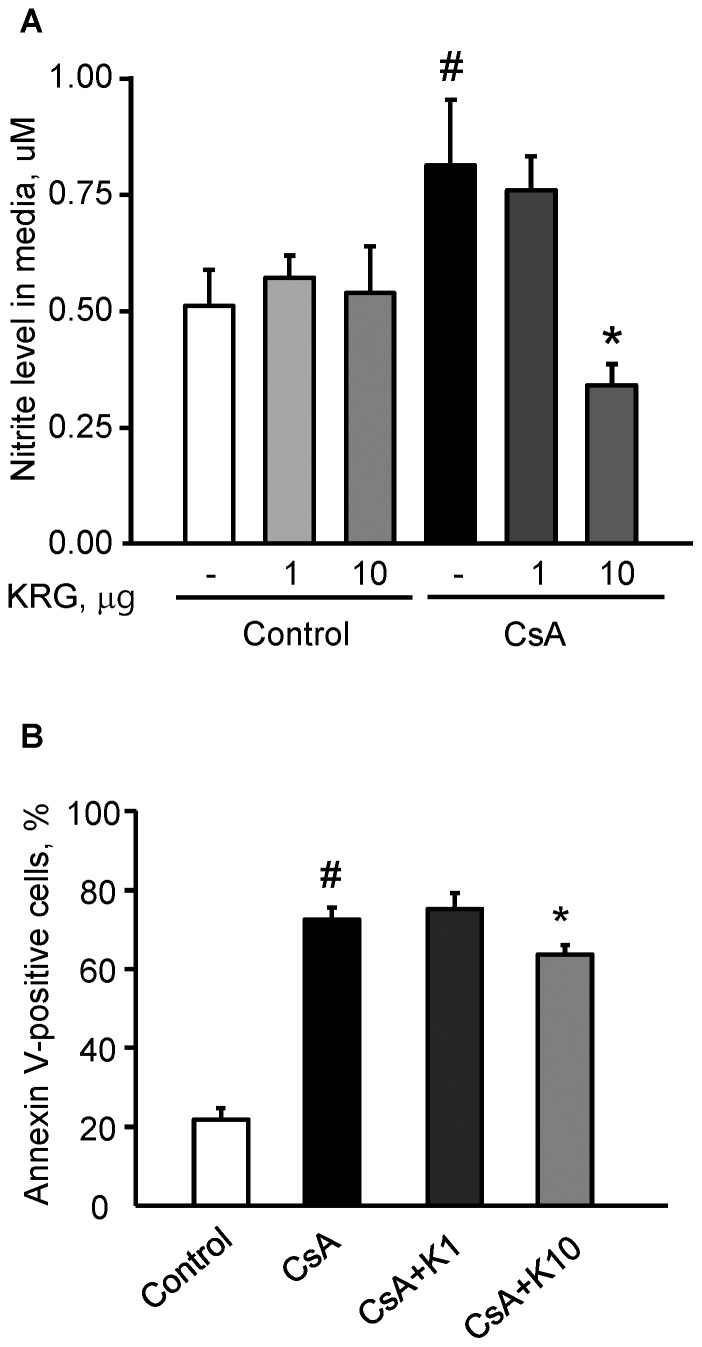
Effect of KRG on apoptotic cell death in INS-1 cells during CsA-induced injury. (A and B) TUNEL staining was used to detect apoptosis, and its quantitative analysis is shown for INS-1 cells treated with either CsA alone or CsA plus 1 or 10 µg/mL of KRG for 24 h. TUNEL-positive nuclei (arrows) were rarely observed in the control group or in those treated with KRG alone. However, the numbers of TUNEL-positive nuclei increased significantly with CsA treatment compared with the control, and the addition of KRG decreased this. (C) Immunoblot analyses for Bcl-2, Bax and active caspase-3 in the INS-1 cells. The expression of Bcl-2 was reduced significantly in both CsA plus K groups compared with the CsA-alone group. By contrast, the expression levels of Bax and active caspase-3 were reduced in both CsA plus K groups compared with the CsA-only group. Magnifications×400. Relative optical densities of bands in each lane were normalized with each β-actin band from the same gel. ^#^P<0.05 vs. Control or K1 or K10; *P<0.05 vs. CsA.

To investigate the underlying molecular mechanisms of the antiapoptotic effect of KRG in CsA-induced injury, INS-1 cells were treated with CsA alone or combined with KRG (1 or 10 µg/mL) for 24 h. Figures 7A and 7B show TUNEL staining and its quantitative analysis in the four groups. The number of TUNEL-positive cells increased significantly with CsA treatment compared with the control, but the addition of KRG decreased them (Control, 1.1±0.3; K10, 0.9±0.2; CsA, 35.7±5.2; CsA+K10, 18.2±2.3; CsA vs. control or K10; CsA vs. CsA+K10, P<0.05). Figure 7C shows that CsA suppressed expression of the antiapoptotic marker Bcl-2 but that this suppression was attenuated by cotreatment with KRG. CsA treatment induced the expression levels of the proapoptotic markers Bax and active caspase-3, and these were attenuated by cotreatment with KRG. Thus, KRG had antiapoptotic effects in INS-1 cells during CsA treatment.

### Effect of KRG on CsA-induced Oxidative Stress in INS-1 Cells

**Figure 8:**
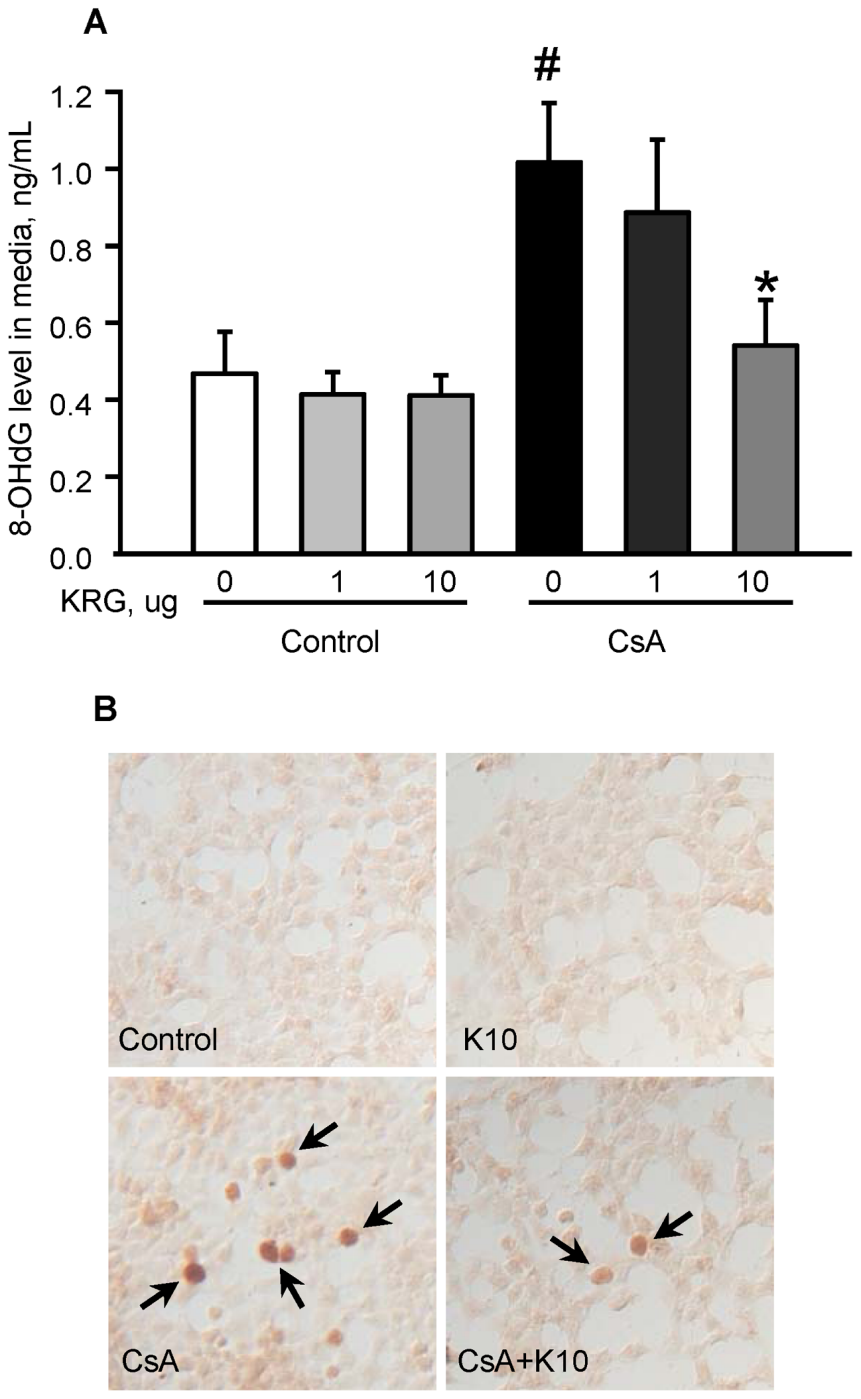
Effect of KRG on CsA-induced oxidative stress in INS-1 cells. (A) Graph showing 8-OHdG levels in culture media after treatment with CsA or CsA plus 1 or 10 µg/mL of KRG for 24 h. The addition of 10 µg/mL of KRG during CsA treatment significantly decreased 8-OHdG production. (B) Immunostaining for 8-OHdG after treatment with CsA or CsA plus 10 µg/mL of KRG for 24 h. The numbers of 8-OHdG-positive nuclei (arrows) increased with CsA treatment compared with control or KRG treatment alone and the addition of KRG decreased the numbers of 8-OHdG-positive nuclei. Magnifications×400. ^#^P<0.05 vs. control; *P<0.05 vs. CsA.

To evaluate the effect of KRG on the induction of oxidative stress markers by CsA treatment in a cell culture setting, we measured the 8-OHdG levels in the culture medium and in INS-1 cells treated with CsA with or without KRG. As shown in Figure 8A, the 8-OHdG level in the medium from CsA-treated cells was significantly higher than that from untreated cells, but this increase was significantly inhibited by cotreatment with 10 µg/mL of KRG (0.47±0.1 ng/mL in the control group vs. 1.0±0.2 ng/mL in the CsA group; 0.54±0.1 ng/mL in the CsA+K 10 µg/mL vs. 1.0±0.2 ng/mL in the CsA, P<0.05). Immunostaining for 8-OHdG revealed that the number of 8-OHdG-positive nuclei increased markedly and that the addition of KRG reduced them (Figure 8B). Thus, consistent with our in vivo findings, cotreatment with KRG during CsA treatment protected INS-1 cells from inflammation and apoptotic cell death by decreasing CsA-induced oxidative stress.

## Discussion

The current study was conducted to investigate the protective effect of KRG against CsA-induced pancreatic β cell injury in a mouse model and in INS-1 cell cultures. The protective effect of KRG was demonstrated by a recovery of insulin, blood glucose levels, and by reduction in inflammation and apoptotic cell death following chronic CsA treatment. Furthermore, the antioxidative properties of KRG were associated with a delay in the progression of CsA-induced morphological and functional β cell impairment by reducing oxidative stress. Our results provide a rationale for the use of KRG as a potential supplemental treatment to attenuate CsA-induced pancreatic β cell injury in transplant recipients.

Injury of pancreatic β cells by CsA has been reported to lead to a disturbance in glucose metabolism and decreased insulin gene transcription, synthesis, and secretion [22], [23]. These findings are consistent with our results, which indicate that the AUCg based on IPGTT was increased and that the serum insulin level and pancreatic insulin immunoreactivity were decreased by chronic CsA treatment. However, cotreatment with KRG significantly decreased the AUCg and increased the serum insulin level compared with treatment with CsA alone. Furthermore, immunohistochemical staining for insulin revealed that compared with treatment with CsA alone, KRG cotreatment resulted in increased pancreatic islet size, greater insulin immunoreactivity, and well-preserved pancreatic islet morphology with less irregular islet boundaries and reduced vacuolization. These findings suggest that KRG treatment has beneficial effects on the preservation of islet β cells against CsA-induced damage, resulting in the preservation of insulin content and normalization of blood glucose levels. With regard to the combination with CsA, KRG treatment attenuated the pancreatic dysfunction and islet cell damage as described above. Interestingly, the VH+K0.4 group showed significantly lower glucose tolerance than VH group even though there were no significant differences of parameters such as insulin level, islet size and basic parameters. This finding suggests that the use of large amounts of ginseng may be harmful in healthy individuals. Overall, our study revealed beneficial effect of ginseng in treating CsA-induced islet dysfunction.

The pathogenesis of pancreatic β cell damage induced by CsA has been suggested to result in part from inflammatory processes. Indeed, recent studies have demonstrated that anti-inflammatory agents such as lisofylline are beneficial in attenuating or preventing disease [24]–[26]. Both clinical and basic research studies demonstrate that immune cells and inflammatory mediators, including cytokines, play vital roles in the pathogenesis of diabetes and its complications. However, it is not clear whether pancreatic β cell injury induced by chronic CsA treatment is associated with immune cell infiltration and cytokine production. Our results clearly showed that chronic CsA treatment significantly increased not only the number of macrophages infiltrating islets (by about 1.5-fold) but also the level of iNOS and proinflammatory cytokines in pancreatic β cells. CsA treatment also induced significantly increased levels of nitrite, an indicator of NO production, in the culture medium from INS-1 cells. When KRG treatment is combined with CsA treatment in vivo, we predict that KRG will protect pancreatic β cells against the CsA-induced inflammatory microenvironment described above. In fact, several studies have demonstrated that KRG inhibits the production of inflammatory cytokines and activation of NFκB, suggesting that it has an anti-inflammatory effect [27], [28]. The results of our *in vivo* study show that oral administration of KRG reduced CsA-induced macrophage infiltration and the levels of iNOS and cytokines (e.g., IL-6 and IL-17) in pancreatic β cells. Our in vitro study using INS-1 cells showed that KRG treatment also decreased CsA-induced NO production. These findings suggest that KRG exerts its anti-inflammatory effects through the reduction of macrophage migration to the injured area, resulting in a reduction in the production of CsA-induced iNOS and proinflammatory cytokines. These properties of KRG, acting either directly or through inhibition of inflammation, could be useful in transplant recipients with chronic CsA toxicity.

Apoptosis is a major type of cell death in CsA-induced organ toxicity. We previously reported that CsA-induced renal injury is closely associated with activation of apoptosis-related genes, and fibrotic tissue replaces the apoptotic cells in chronic CsA-induced nephropathy [9], [17]. At molecular basis, we also reported that CsA induces Bax aggregation and translocation to the mitochondria, causing activation of caspase-9, which then cleaves and activates the effector caspase: caspase-3. Activation of caspase-3 leads to a loss of mitochondrial transmembrane potential and to apoptotic cell death [29]. In this study, histological evidence of apoptosis-as assessed by the TUNEL assay-showed that CsA-treated pancreatic β cells were significantly more likely to undergo apoptosis than were untreated cells. Consistent with this, CsA treatment increased the expression of Bax and active caspase-3, but downregulated expression of the antiapoptotic protein Bcl-2. These changes were attenuated by cotreatment with KRG both in vivo and in vitro. Notably, the ratio of Bcl-2 to Bax, which is correlated with cell survival following an apoptotic stimulus, also returned to the normal level when KRG was cotreated with CsA [30]. Based on these findings, we speculate that dietary KRG might play an important role in the balance between antiapoptotic and proapoptotic signaling, thereby contributing to improvements in pancreatic β cell function. These antiapoptotic properties of KRG are thought to be mediated by a reduction in the intra-islet inflammatory mediators that can trigger a final common pathway leading to apoptosis. The proapoptotic effect of ginseng was also reported in study using basilar artery smooth muscle cells and HeLa cells. In their studies, ginseng exerted proapoptotic effect to prevent cerebral vascular remodeling during the development of hypertension [31], and progression of human cervical cancer [32]. Thus, enhanced apoptotic cell death by ginseng can be interpreted as being protective, by combating hypertension and cancer. In our study, ginseng has antiapoptotic properties in pancreatic islet β cell, and this was beneficial in reducing CsA-induced pancreatic islet β cell dysfunction. Taken together, we suggest that ginseng exerts protective effect by inducing anti-or pro-apoptotic cell death according to different pathologic conditions.

Next, we evaluated reactive oxygen species (ROS) scavenging by KRG to counter CsA-induced oxidative stress, a common pathway of CsA-induced organ injury [9], [33]. For this, we measured the expression of 8-OHdG, a marker of oxidative DNA damage, both in vivo and in vitro. The results showed that the expression of 8-OHdG was increased in the serum and pancreatic β cells of CsA-treated mice and in the culture medium from CsA-treated INS-1 cells but that cotreatment with KRG attenuated these increases. These results are consistent with our data for pancreatic β cell function, morphology, inflammation, and apoptotic cell death when CsA treatment is combined with KRG. Indeed, there is increasing evidence to show that the development of diabetes is associated with increased oxidative stress, and that KRG or other types of ginseng can scavenge ROS with a resultant antihyperglycemic effect [13], [34], [35]. Overall, our results indicate that orally administered KRG has significant antihyperglycemic activity and might effectively reduce CsA-induced pancreatic β cell damage.

**Figure 9:**
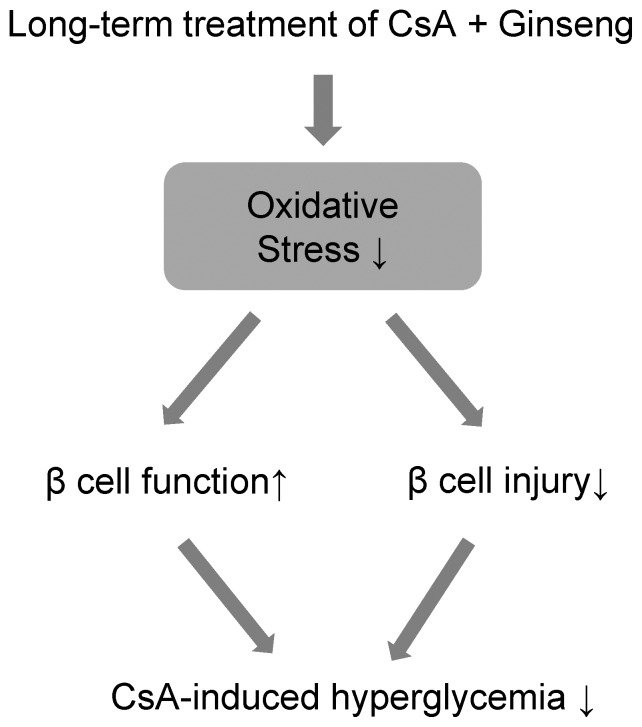
Diagram of the proposed mechanism of the protective effect of KRG.

CsA is a diabetogenic agent when its use is prolonged [36] Indeed, posttransplant diabetes mellitus (PTDM) induced by CsA treatment is a frequent and serious complication after solid organ transplantation, and is considered to be a type of secondary diabetes mellitus involving impaired insulin secretion and insulin resistance [2]. Although information about this condition is increasing, the ultimate mechanisms underlying CsA-induced diabetes and related complications are not completely understood. In this study, we found that a high level of CsA-induced oxidative cellular damage was closely associated with hyperglycemia (Figure 9). As a secondary effect, this high-glucose condition is known to aggravate ROS production (or to result in inadequate elimination) and can cause chronic diabetes [37]–[40]. We suggest that the main cause of diabetes in transplant recipients receiving CsA is oxidative stress, because the effects of CsA were corrected by a strong anti-ROS agent: the KRG extract. Thus, dietary supply of an antioxidant such as KRG might reduce or delay pancreatic β cell dysfunction and injury in CsA-induced diabetes by providing protection against oxidative damage.

Our experimental model of chronic CsA pancreatic toxicity had some limitations. The basal CsA levels were much higher than those targeted in clinical practice. This higher CsA level is species specific. Unlike humans, mice are resistant to CsA-induced injury. Therefore, salt depletion combined with high-dose CsA treatment is needed to induce the functional impairment and morphologic consequences of CsA [6], [9], [16], [41], [42]. A follow-up period longer than 4 weeks might be preferable to mimic chronic disease, but the study period could not be extended because mice cannot survive beyond 4 weeks under treatment with CsA (30 mg/kg of body weight) and a low-salt diet.

In this study, we used red ginseng extract rather than specific ginsenosides because this is one of the most popular ways for humans to consume ginseng. It would be valuable to study the functions of specific constituents of ginseng because ginseng extracts contain multiple functional constituents.

In conclusion, we have demonstrated that prolonged oxidative injury induced by CsA treatment led to severe pancreatic β cell injury and dysfunction in vivo, and that the oral administration of ginseng extract showed protective effects by decreasing oxidative stress. These beneficial effects of ginseng could be helpful to a delay in the onset of CsA-induced diabetes. Our results provide good evidence that KRG has significant potential as a therapeutic intervention in patients with immunosuppressant-associated diabetes.
